# Co-infection of pigs with *Taenia solium* cysticercosis and gastrointestinal parasites in Eastern and Western Uganda

**DOI:** 10.1007/s00436-021-07380-9

**Published:** 2021-11-24

**Authors:** Nicholas Ngwili, Lian Thomas, Samuel Githigia, Dishon Muloi, Karen Marshall, Raphael Wahome, Kristina Roesel

**Affiliations:** 1 Animal and Human Health Program, International Livestock Research Institute, Nairobi, Kenya; 2 Faculty of Veterinary Medicine, University of Nairobi, Nairobi, Kenya; 3 Institute of Infection, Veterinary and Ecological Sciences, University of Liverpool, Leahurst Campus, Neston, CH64 7TE UK; 4 Institute for Parasitology and Tropical Veterinary Medicine, Freie Universität Berlin, Berlin, Germany

**Keywords:** *Taenia solium*, Porcine cysticercosis, Gastrointestinal parasites, Co-infection

## Abstract

A study was carried out in Kamuli and Hoima districts in Eastern and Western regions of Uganda to determine the *Taenia solium* porcine cysticercosis (PCC) and gastrointestinal (GI) parasites co-infection status in pigs. One hundred sixty-one households were selected randomly and visited between November and December 2019. A household questionnaire was administered, and faecal and blood samples were collected from at least one pig older than 3 months per household. A blood sample was obtained from a jugular venipuncture, and a rectal faecal sample was obtained. *Taenia* spp. circulating antigen levels in the sample sera were tested using a commercial enzyme-linked immunosorbent assay kit, apDia™ cysticercosis Ag ELISA. The modified McMaster technique was used to identify and quantify the GI parasites. The apparent animal-level seroprevalence for PCC was 4.8% (95% CI 2.7–7.1) and differed across the two districts (*p* = 0.018). At the pig herd level, the prevalence was 9.7% (95% CI 5.5–14.4). The prevalence of the different nematode eggs and coccidian oocysts in the two districts was as follows: strongyles 79.0% (95% CI 74.3–83.6), coccidia 73.3% (95% CI 68.3–78.6), *Trichuris* spp. 7.4% (95% CI 4.9–10.6), *Strongyloides ransomi* 2.1 (95% CI 0.7–3.5) and *Ascaris* spp. 4.9 (95% CI 2.8–7.4). Overall, across the two districts, the arithmetic mean for the oocysts per gram (OPG) for coccidia was 2042.2 ± 5776.1, and eggs per gram (EPG) were the highest in strongyles 616.1 ± 991. Overall, 57.4% of the porcine cysticercosis seropositive pigs were also positive for at least one of the gastrointestinal helminths which included strongyles, *Strongyloides ransomi*, *Trichuris* spp. and *Ascaris* spp. The co-infection status of pigs with both PCC and GI parasites demonstrated by this study can provide an incentive for integrating the control and management of both parasites with oxfendazole. Further studies are required to understand the feasibility of using oxfendazole including cost–benefit analysis and the acceptability by local stakeholders for the control of *T. solium* cysticercosis and gastrointestinal parasites in pigs.

**Supplementary Information:**

The online version contains supplementary material available at 10.1007/s00436-021-07380-9.

## Introduction

In Uganda, the production and consumption of livestock and livestock products have been growing rapidly, with the greatest growth observed in the pig sector (Twine and Njehu 2020). The establishment of piggeries and increased pig production by rural farmers is encouraged by the government and forms part of the central government agricultural plan (Waiswa et al. 2009). The pig sector is generally underdeveloped, although it has high growth potential, given the rising demand for pork domestically and in neighbouring countries such as South Sudan, Rwanda and the Democratic Republic of Congo (Ouma et al., 2017). Uganda has the highest per capita pork consumption in the East African region at 3.4 kg per year (FAOSTAT 2021). The pig enterprise supports the livelihoods of up to over 1.1 million households (MAAIF and UBOS 2008). Pigs are preferred over other livestock species due to their high reproduction rate with high fecundity per reproduction cycle. This makes pig production important for income generation to meet different household financial needs (Ouma et al. 2015). Additionally, pigs act as a source of saving/ ‘piggy bank’ to be sold in times of financial distress, mostly to pay school fees and hospital bills (Ouma et al., 2015, 2014).

Despite the pig production enterprise being an important source of livelihood for the rural smallholder farmers in Uganda, animal health issues including *Taenia* (*T.*) *solium* cysticercosis, gastrointestinal parasites and African Swine fever (ASF) pose a significant risk to the growth of the sector and negatively impact on pig and human health (Dione et al. 2014). Porcine cysticercosis, caused by the larval stage of *T. solium*, does not cause productivity constraints but contributes to perpetuating the parasite’s life cycle, which causes severe neurological disease in humans in endemic settings. Pigs are the intermediate host for *T. solium*. They acquire the infection through ingesting viable eggs, excreted during defecation by a human infested by the adult stage of the tapeworm as the pig scavenges. The eggs may also contaminate feed, water and the environment (García et al. 2003). This parasite is endemic in sub-Saharan Africa imposing an ever-increasing human health and economic burden with increased consumption of pork (Zoli et al. 2003; Havelaar et al. 2015). The parasite thrives in these settings due to the increasing popularity of raising pigs mostly under an extensive system of production coupled with poor sanitary conditions (Phiri et al. 2003; Braae et al., 2015b).

Despite the availability of proven simple ways to break the *T. solium* life cycle, the parasite remains largely uncontrolled in Uganda. The lack of control appears to be due to a lack of awareness of the public health implications and competing priorities with other gastrointestinal parasites and ASF that directly impact farmers’ income and livelihood (Kungu et al. 2015). While *T. solium* is a parasite of public health importance, GI parasites impose an additional, day-to-day chronic burden on pig keeping households through reduced productivity. At the same time, some are zoonotic and of public health importance (Nejsum et al. 2012). Pig farmers acknowledge the need to control GI parasites through deworming to improve growth and weight gain (Dione et al. 2014; Thompson 2017). This could provide an opportunity to integrate the control of GI parasites with the control of PCC.

The most prevalent gastrointestinal helminths in Africa and reported in Uganda are from the following taxa: *Ascaris* spp., *Strongyloides* spp., strongyles (*Oesophagostomum dentatum* and *Hyostrongylus rubidus*), *Trichuris* spp. and coccidia (Nissen et al. 2011; Roesel et al. 2017)*.* Infection of pigs with these parasites may reduce daily feed intake, weight gain, feed conversion efficiency and overall carcass quality (Kipper et al. 2011; Knecht et al. 2011; Ózsvári 2018). In addition, the condemnation of livers resulting from ascariasis and mortality in piglets due to coccidia infections can reduce the pig enterprise’s profitability (Ózsvári 2018). *Ascaris suum* and *Trichuris suis* are also known to have zoonotic potential and are therefore of public health concern (Nejsum et al. 2012).

The control of both *T. solium* cysticercosis and GI parasites in pigs requires changes in pig husbandry practices to avert the risk of exposure to infective materials and the judicious administration of anthelmintic drugs. The administration of oxfendazole at 30 mg per kg effectively kills *T. solium* cysts and has also been demonstrated to control *A. suum*, *Oesophagostomum* spp*.*, *T. suis* and *Metastrongylus* spp. (Alvarez et al. 2013; Mkupasi et al. 2013a). Integrating such strategies as oxfendazole dosing with health education, improved pig housing and feeding may support *T. solium* cysticercosis control and at the same time improve pig production profitability through the control of pig GI parasites. The objectives of the current study were to (i) to estimate the prevalence of PCC, gastrointestinal parasites and the level of co-infection in pigs, (ii) to assess the risk factors associated with the infection with PCC (iii) and to provide the evidence base to support integrated control of pig gastrointestinal parasites as an integral aspect of *T. solium* cysticercosis control.

## Materials and methods

### The study area

A cross-sectional survey was conducted in Kamuli and Hoima districts Uganda from November to December 2019. Uganda is a landlocked country located in East Africa and lies across the equator, about 800 km inland from the Indian Ocean. It has a landmass of 200,523 km^2^ of the total area of 241,551 km^2^ (Uganda Bureau of Statistics 2016). Uganda is divided into districts, counties, sub-counties, parishes and villages (Uganda Bureau of Statistics 2018). Kamuli district is in the lowland areas of eastern Uganda at an average altitude of 1066 m above sea level. Hoima district is in the Northwest at an average altitude of 1122 m above sea level and has bimodal rainfall distribution with long and short rain seasons. These districts have high proportions of pig rearing households (5–17% and 30–42% for Kamuli and Hoima district, respectively). In terms of pig numbers, Hoima has 104,669 pigs and Kamuli district 55,239 pigs (MAAIF and UBOS 2008) and has a high demand for pig meat and pig products (Ouma et al., 2017).

### Study site and household selection

The study focused on the districts of Kamuli and Hoima, which were sites for the International Livestock Research Institute (ILRI) led by Uganda Pig Genetics project (UPG), which formed the basis for entry to the study sites for the current study. The districts have also been a focus for ILRI’s research on smallholder pig value chain, and the initial selection has been described elsewhere (Dione et al. 2014; Ouma et al. 2015). A recent study indicated a high prevalence of PCC in pigs in Kamuli district (Kungu et al. 2017b), but no study has been undertaken in Hoima district. Five and three sub-counties were purposively selected in Kamuli and Hoima district, respectively, based on the pig population densities. Five parishes were selected under each sub-county depending on the number of pig rearing households.

**Fig. 1:**
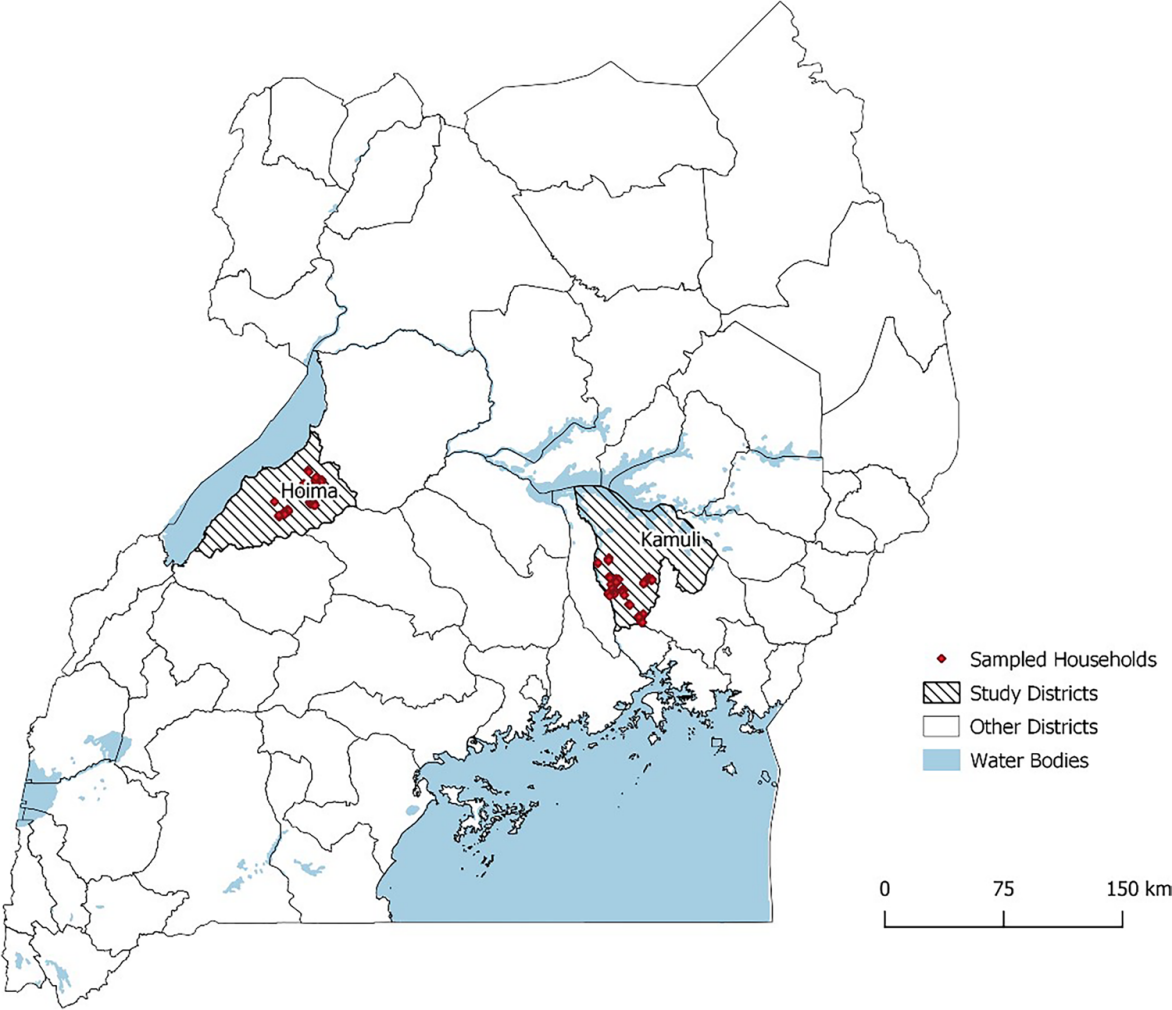
Map of Uganda showing the study sites (shaded) and sampled households (red dots)

Further, three villages with the highest number of pig rearing households were selected from each of the chosen parishes. Thus, a total of 30 villages were included in the study across the two districts. In each of the 30 selected villages, a random sample of 7 households was drawn from the list of households generated by the veterinary officials in the district to target 200 households (Fig. 1).

### Sample size calculation

For the estimation of the prevalence of PCC, the sample size calculation was premised on the expected herd prevalence of PCC of 25.7% (Nsadha et al. 2014) using the formula, *n* = [Z^2^Pq]/d^2^, where *n* is the required sample size; *Z* is the multiplier from a standard normal distribution (1.96) at a probability level of 0.05; *P* is the estimated prevalence of 25.7%; *q* is (1 − *P*) that is the probability of having no disease; and *d* is the desired precision level (5%). This formula gives a sample size of *n* = 293 pigs as the minimum. For gastrointestinal parasites, the same formula as above is used, and the expected prevalence of 61% (Roesel et al. 2017) and precision of 5% are used. The sample size was estimated to be 365 as the minimum.

### Collection of household data

A household-level questionnaire was pretested, and changes were incorporated in the final version. The questionnaire was administered using Open Data Kit (ODK) to the household head, spouse or the person familiar with the running of the pig enterprise. Data were collected on self-reported and observational variables: pig confinement (during the rainy and dry season and during the day and night); pig water source during the dry and wet season; self-reported pig health management practices including deworming; pork slaughter and consumption practices; household hygiene and deworming practices; knowledge on *T. solium* infection; and toilet availability and signs of frequent use, e.g. a clear path to the toilet, complete wall and door.

### Pig blood and faecal sampling

Sensitization meetings were conducted at the village level a week prior to start of the survey. During the sensitization meeting, the project team introduced the project, and written informed consent was obtained from the farmers. During the household visits, pigs that were 3 months or older, not pregnant, not lactating and not manifesting overt clinical signs were sampled up to a maximum of 3 pigs per herd. If the farm had more than 3 eligible pigs, a serial number was allocated to the pigs, and 3 pigs were selected using random numbers generated by a random number generator app running on a mobile device (https://play.google.com/store/apps/details?id = ru.uxapps.random&hl = en&gl = US).

Pigs were restrained using a pig snare or held in dorsal recumbency if under approximately 10 kg. Registered veterinary officers collected blood from the anterior vena cava on the pig’s right side (Zimmerman et al. 2012) into a single BD Vacutainer® 10-ml plain tube labelled with the household and individual animal ear tag number. In addition, a faecal sample was collected from the rectum while the pig was restrained using the standard pig snare and samples were placed in BD Falcon™ 50-ml conical tubes labelled with the animal ear tag numbers. The pigs were then weighed by corralling the pig into a cage mounted on an electronic weighing scale.

The blood samples were centrifuged at 3000 rpm for 20 min at room temperature at the field laboratory. Obtained sera were then separated into two aliquots in 2-ml cryovials labelled with a unique barcode. Faecal samples were transported on ice to the Central Diagnostics Laboratory (CDL) at Makerere University in Kampala for processing and analysis within 24 h after collection. Serum samples were stored temporarily in the field at − 20 °C and transported to CDL after 2 weeks for analysis.

### Serology

*Taenia* spp. circulating antigen in porcine sera was tested using a commercial enzyme-linked immunosorbent assay kit, apDia cysticercosis Ag ELISA (ApDia 2019), following the manufacturer’s instructions. The tests were run in duplicate, and optical densities (ODs) of samples were measured at 450 nm with a reference wavelength of 630 using a microplate reader (Biochrom®, Cambridge – CB4 0FJ, England) with the cut-off value calculated per plate as the mean OD of the negative control multiplied by 3.5. The antigen index (Ag index) of each sample was calculated by dividing the OD value of the sample by the cut-off value. The cut-off value was calculated per the manufacturer’s instructions using the mean OD of the negative control provided in the kit. As recommended by the manufacturer, animals were classified as negative if the Ag index was ≤ 0.8 and positive if ≥ 1.3. The ELISA test used has a sensitivity of 86.7% and specificity of 94.7% (Dorny et al. 2004).

### Gastrointestinal parasite identification and counts

As described by Zajac and Conboy (2007), the modified McMaster technique was used to identify and quantify coccidia oocysts, strongyles, *Strongyloides* spp., *Trichuris* spp. and *Ascaris* spp*.* eggs. Briefly, 4 g of faecal material was weighed and mixed with 56 ml of a saturated common salt solution. The mixture was thoroughly stirred and filtered using a tea strainer (mesh size 500–800 µm). The filtrate was stirred with a Pasteur pipette, and a sub-sample was picked while stirring and transferred to the first chamber of the McMaster slide while ensuring no air bubbles were left on the slide. While still stirring, the other chamber of the slide was filled, and the McMaster slide was left to stand for 5–10 min to allow the eggs to float. The McMaster slide was examined using the compound microscope at 10 × 10 magnification, and the eggs within each grid were identified and counted. To get the total number of each type of worm eggs, the number obtained per species was multiplied by 50 to obtain the eggs per gram (EPG) and oocysts per gram (OPG) for coccidia. The results were recorded in Microsoft Excel version 2016. The method used has an estimated sensitivity of 66.67% and specificity of 81.06% (Scare et al. 2017).

### Data management and analysis

Data was collected from 161 households in both Kamuli and Hoima districts using a semi-structured questionnaire developed and administered in ODK. After cleaning and merging, 144 records were complete with blood and faecal sample and a corresponding sample metadata form. A dichotomous outcome variable was computed as the presence or absence of PCC and GI parasite as either being positive or negative by Ag ELISA and Mc Master slide technique, respectively, to determine prevalence.

Descriptive statistics, including the prevalence levels of PCC and GI parasites and their 95% confidence intervals, were calculated using the *DescTools* and *gmodels* package (Warnes et al. 2018; Signorell 2020). Differences in the proportions of the different variables in the two districts were tested using Fisher’s exact test and *p* values reported. Both univariable and multivariable analyses were done using the *glmer* function of the *lme4* package in R (Bates 2010) with village (village ID) as a random effect to account for clustering in the sampling design.

**Fig. 2:**
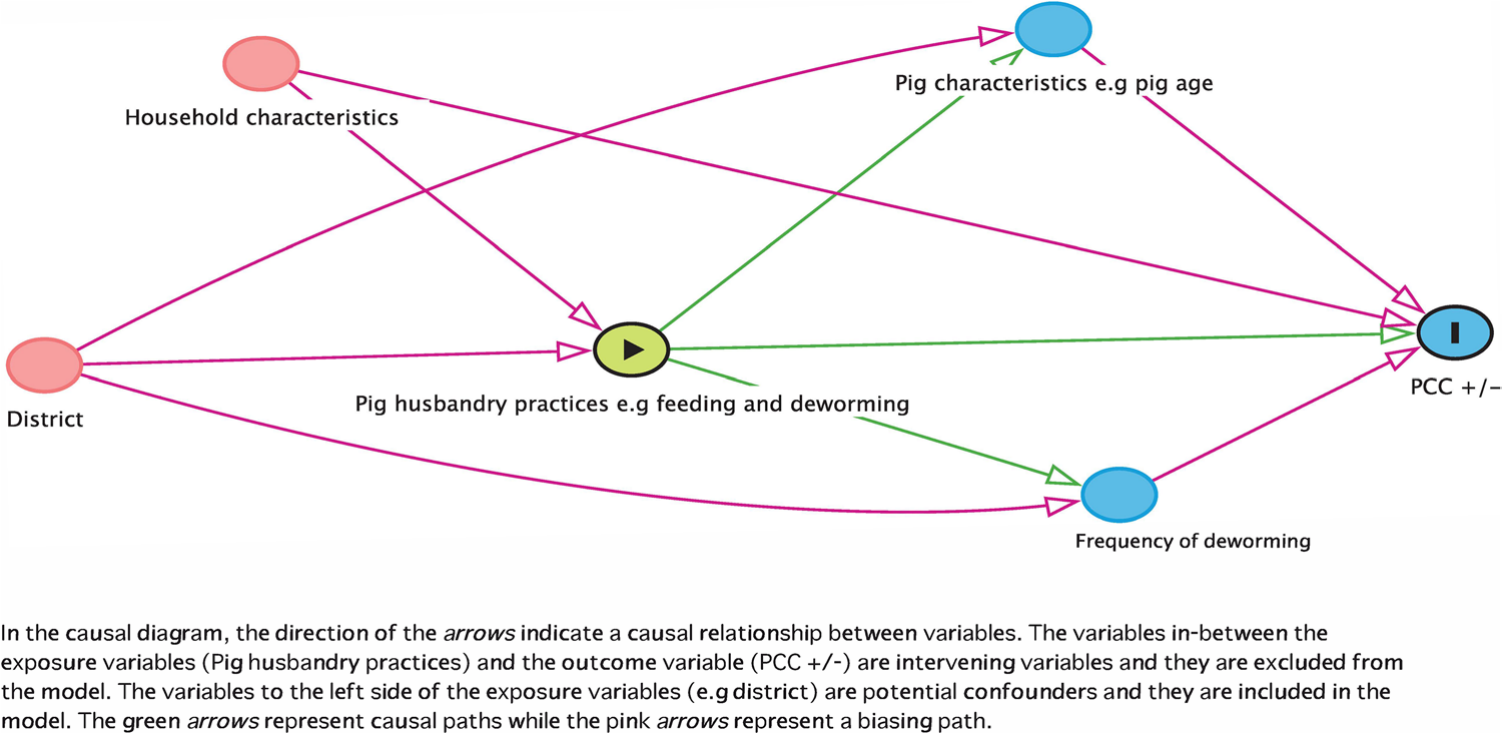
A causal model diagram showing the potential association of exposure variables and the outcome variable (positive or negative PCC)

As an initial step in selecting the potential predictors for PCC seropositivity, independent variables were tested for correlation using Pearson’s chi-square and Fisher Exact test, eliminating those closely correlated at *p* < 0.05. Secondly, the unconditional association was tested using Fisher’ exact test to reduce the number of variables. Over 30 independent variables were tested; they included variables on pig confinement, feeding, pig water sources, deworming, pork consumption and knowledge variables. Additionally, causal diagrams or directed acyclic graphs (DAGs) were constructed in Dagitty online platform (Textor et al. 2011) to postulate the relation between potential predictors and the outcome variable (Fig. 2). In the causal diagram, pig husbandry practices were directly associated with the disease status, with pig characteristics and frequency of deworming considered intervening variables. Household characteristics that encompass variables such as farmers’ education and knowledge levels may also influence the outcome. In the causal diagram, we hypothesized that having knowledge on *T. solium* transmission under the household characteristics would have a protective effect on seropositivity.

The association of predictor variables and PCC seropositivity (negative/positive) was tested at the household level using univariable logistic regression. The models were not built at the individual level because only two variables were measured at the animal level, age and weight and they were highly correlated. Predictors with *p* < 0.1 were retained and used to fit a generalized linear mixed effects model (GLMM) with village as a random effect. All tests for significance were performed at the *α* = 0.05 level, and odds ratios (OR) and 95% confidence intervals (CI) were determined. The models were built by first having a global model with all potential predictors identified through the univariable modelling. The final model was then selected automatically based on information theory using the *dredge* function in *MuMIn* package in R (Barto 2020). This methodology compares Akaike information criterion (AIC) for the different models and selects the one with the least AIC. Finally, a GLMM (with village as a random effect) of the top model from the dredge analysis was fitted, and OR and confidence intervals were calculated using sjPlot package in R.

## Results

### Descriptive analysis results

Demographic characteristics of the study population, pig husbandry practices and household hygienic practices are summarized in Tables 1, 2 and 3, respectively. The majority of respondents interviewed were female (67.9% in Kamuli and 57.6% in Hoima district), and secondary education was the commonest level of education (67.1% in Kamuli and 59.7% in Hoima district) (Table 1). Blood and faecal samples were obtained from 294 pigs, the majority female pigs from 144 households in both Kamuli and Hoima districts. The average herd size for the two districts including piglets was 5 pigs (range, 1–41). The average weight and age of the sampled pigs was 22.13 kg (range 7.2–111) and 7.4 months (range 3–39), respectively. Most sampled pigs were crossbreeds between local and exotic breeds (52.4% in Kamuli and 100% in Hoima district) as determined by the farmer and research team. A recent study has shown mix of old British and modern pig ancestries in the crossbreeds found in the study areas (Babigumira et al. 2021).

**Table 1:** Demographic characteristics

Demographic characteristic	Kamuli % *n* = 78	Hoima % *n* = 66
Sex of respondent		
Male	32.1	42.4
Female	67.9	57.6
Level of education		
Primary	6.6	0.0
Secondary	67.1	59.7
Vocational school	25.0	24.2
Technical/diploma	0.0	3.2
University	1.3	11.3
Other	0.0	1.6

**Table 2:** Pig husbandry practices

Pig husbandry practices	Kamuli, %	Hoima, %
Confinement of piglets		
Free ranging	27.3	27.5
Tethered	18.2	71.1
Housed	54.5	1.3
Confinement of growers		
Free ranging	2.8	26.3
Tethered	60.6	21.1
Housed	36.6	52.6
Confinement of sows		
Free ranging	0.0	1.7
Tethered	58.3	71.2
Housed	41.7	27.1
Confinement of boars		
Free ranging	0.0	4.2
Tethered	69.2	66.7
Housed	30.8	29.2
Feed use		
Feeding pigs on maize bran		
Yes	96.2	74.2
No	3.8	25.8
Feeding pigs with sweet potato vines		
Yes	44.9	87.9
No	55.1	12.1
Feeding pigs on unboiled swill		
Yes	56.4	57.6
No	43.6	42.4
Feeding pigs on pigweed (*Amaranthus spp.)*		
Yes	6.4	12.1
No	93.6	87.9
Feeding pigs on yam leaves		
Yes	3.8	87.9
No	96.2	12.1
Shallow well		
Yes	87.0	59.1
No	13.0	40.9
Rainwater		
Yes	57.4	6.1
No	42.6	93.9
Deworming pigs		
Yes	97.3	98.5
No	2.7	1.5
Type of anthelmintic used in pigs		
Albendazole	48.7	1.5
Levamisole	16.7	90.9
Ivermectin	10.3	0.0
I don’t know	17.9	0.0
Not dewormed	6.4	7.6
Frequency of deworming pigs		
Never dewormed	14.1	6.1
At 3-month interval	73.1	74.2
More than 3-month interval	11.5	0.0
Other^a^	1.3	19.7

Other^a^, 2 months, depending on whether pig looks sick, when the vet visits

**Table 3:** Household hygienic practices

Household hygienic practices	Kamuli %, *n* = 78	Hoima %, *n* = 66
Last time respondent dewormed		
Never dewormed	16.7	45.5
At 3-month interval	19.2	13.6
Once a month	5.1	12.1
I cannot remember	57.7	6.1
Others^a^	1.3	22.7
Deworming of children in school		
Yes	58.8	92.7
No	41.2	7.3
Boiling of drinking water		
Yes	10.3	25.8
No	89.7	74.2
Source of drinking water		
Pipe water to the house	0.0	1.5
Pipe water to the compound	1.3	3.1
Public tap	0.0	9.2
Shallow well	96.2	72.3
Surface water	2.6	7.7
Natural spring	0.0	1.5
Rainwater	0.0	4.6
Presence of latrine		
Yes	92.3	100
No	7.7	0.0
Signs of toilet use		
Path to toilet		
Yes	89.7	98.5
No	10.3	1.5
Complete wall/door		
Yes	42.3	34.8
No	57.7	65.2

Other^a^, every 6 months, annually

The different levels of confinement level of the different pig age categories in the two districts are presented in Table 2. Forty one percent and 27.1% of sows were housed in Kamuli and Hoima districts, respectively. Hoima district had more piglets, growers and sows free ranging than Kamuli district albeit at lower proportions (Table 2). There were significant differences in the types of confinement of the piglets and growers across Kamuli and Hoima district (*p* < 0.05, Fisher’s test).

Sweet potato vines were a commonly used pig feed in both Kamuli and Hoima districts (Table 2). The commonly used source water for watering pigs during both rain and dry season was public shallow wells at 87.0% in Kamuli and 59.1% in Hoima district. In Kamuli and Hoima district, 97.3% and 98.5%, respectively, of farmers indicated that they dewormed their pigs albeit at varying intervals and frequency. Overall, the majority (73.1% in Kamuli and 74.2% in Hoima district) indicated they dewormed the pigs at the 3-month interval (Table 2).

The majority of the surveyed households reported consuming pork either at home (88.20%) or in pork joints (special butcheries in Uganda where pork is roasted and served with vegetables and drinks) (83.85%). Two thirds of people in both districts were aware of human tapeworm infestation, just a half of them had heard of pork cysts or pork measles, and only a handful (6.8%) knew that pigs could get the cysts by eating human faeces. Further, 6.8% and 14.3% of people reported that pigs could acquire cysts from drinking dirty water or from eating contaminated feed, respectively. Only 20% of people in Kamuli had dewormed compared to 45.5% in Hoima, however, only 6.1% of Hoima respondents could remember the date of these last deworming compared to 57.7% of Kamuli residents. However, a large proportion of children had been dewormed under the government mass drug administration (MDA) programs in schools, 58.8% in Kamuli and 92.7% in Hoima district. Among the surveyed households in Hoima and Kamuli district 10.3% and 25.8%, respectively, boiled drinking water. In Kamuli district, 96.2% got drinking water from a public shallow well as compared to 72.3% of the households in Hoima district (Table 3). Overall, 92.3% in Kamuli district and households had a pit latrine. However, 57.7% and 65.2% in Kamuli and Hoima district, respectively, did not have a complete wall and door to provide privacy during use. Overall, it was observed that in 93% of the households, there was a path leading to the toilet showing signs of use.

### Prevalence of porcine cysticercosis

The prevalence of PCC was calculated at both individual and household levels. The apparent individual level seroprevalence was 4.8% (95% CI 2.7–7.1) (Table 4). Individual level PCC seroprevalence differed across the two districts (*p* = 0.017, Fisher exact test). There was no significant difference in exposure to PCC between sex, age and breed of the animal (*p* > 0.05, Fisher exact test). Household-level seroprevalence was 9.7% (95% CI 5.5–14.4) which significantly differed across the study districts 3.8% (1.2–8.2) for Kamuli and 16.6% (9.0–25.6) for Hoima (*p* < 0.001, prop. test).

**Table 4:** Animal-level apparent seroprevalence of porcine cysticercosis, gastrointestinal parasites prevalence and infection intensity

	Kamuli district			Hoima district		Overall	
	% prevalence (95% CI)	Mean EPG/OPG	SD	% prevalence (95% CI)	Mean EPG/OPG	SD	% prevalence (95% CI)
PCC	1.9 (0.6–4.2)	-	-	7.8 (4.2–12.2)	-	-	4.8 (2.7–7.1)
Strongyles	81.9 (76.3–88.3)	684	1078	75.9 (69.3–83.2)	544	889	79.0 (74.3–83.6)
Coccidia *oocysts*^*a*^	69.4 (62.5–77.4)	2418	5987	77.3 (70.8–84.3)	1647	5540	73.3 (68.3–78.6)
*Trichuris* spp.	10.4 (6.2–15.4)	47.2	210	4.3 (2.1–8.0)	17.6	104	7.4 (4.9–10.6)
*Strongyloides* spp.	3.4 (1.3–6.4)	26.7	284	0.7 (0.0–1.9)	2.55	29.9	2.1 (0.7–3.5)
*Ascaris* spp.	6.9 (3.4–10.8)	98.3	529	2.9 (0.7–5.4)	48.2	465	4.9 (2.8–7.4)
*Moniezia* spp*.*	-	0.0	0.0	-	6.57	76.9	
At least one GI parasite infection	93.0 (89.5–97.0)	-	-	90.5 (86.1–95.0)	-	-	91.8 (88.9–94.9)
Polyparasitism	66.6 (59.0–74.3)	-	-	67.8 (60.5–76.1)	-	-	67.2 (61.9–73.0)
Co-infection (PCC + GI parasite)	0.6 (0.0–1.7)	-	-	5.6 (2.8–9.5)	-	-	3.0 (1.3–4.8)
Proportion of co-infection (%)	14.2	-	-	42.8	-	-	57.4

*SD*, standard deviation; *CI*, confidence interval; ^a^*Eimeria* spp. or *Isospora suis*

### Prevalence and infection intensity of pig gastrointestinal helminths

A total of 281 pig faecal samples were examined of which 91.8% (95% CI 88.9–94.9) were positive for GI parasite infections. The arithmetic mean for the oocysts per gram for coccidia was 2418 ± 1078 in Kamuli and 1647 ± 5540 in Hoima district, eggs per gram was highest in strongyles 684 ± 1078 and 544 ± 889 in Kamuli and Hoima districts, respectively, followed by *Ascaris* spp. 98.3 ± 529 in Kamuli and 48.2 ± 465 in Hoima. The EPG was as follows: for *Trichuris* spp. 47.2 ± 210 in Kamuli and 17.6 ± 104 in Hoima district and lastly *Strongyloides* spp. 26.7 ± 284, 2.55 ± 29.9 in Kamuli and Hoima district, respectively. One pig in Hoima district had *Moniezia* eggs (Table 4). There was no difference in the occurrence of the gastrointestinal parasites in the two districts (χ^2^ = 0.31, *p* = 0.5).

The prevalence of the different parasite eggs was as follows: strongyles 79.0% (95% CI 74.3–83.6), coccidia 73.3% (95% CI 68.3–78.6), *Trichuris* spp. 7.4% (95% CI 4.9–10.6), *Strongyloides* spp. 2.1% (95% CI 0.7–3.5) and *Ascaris* spp. 4.9% (95% CI 2.8–7.4) (Table 4). There was a relatively high level of polyparasitism 67.2% (95% CI 61.9–73.0 (Table 4). The level of polyparasitism did not significantly differ across the two districts, breed or sex of the animal (*p* > 0.05, Fisher exact test).

Overall, 57.4% of the proportion of PCC positive pigs were also positive for any of the gastrointestinal parasites, including strongyles, *Strongyloides* spp., *Trichuris* spp. and *Ascaris* spp. excluding coccidia The co-infection proportion was highest for PCC + strongyles (57.1%). The prevalence of co-infection with any GI parasite and PCC was found to be 3.0% (95% CI 1.3–4.8). The co-infection of pigs with any GI parasite and PCC significantly differed across the two districts. Hoima district had more pigs infected by both PCC and gastrointestinal parasites (*p* < 0.05, Fisher exact test). There was no difference in PCC/GI parasite co-infection by sex, breed and age of pig (*p* > 0.05, Fisher exact test).

### Risk factors associated with porcine cysticercosis seropositivity

At the household level, nine variables were tested using univariable model, and five of them were retained for multivariable modelling with *p* < 0.1 (Table 7 in the Supplementary materials). There was a significant association (*p* < 0.05) of household-level seroprevalence of PCC with district and the knowledge that pigs can get infected with PCC by consuming dirty feed at univariate level analysis (Table 5). Furthermore, the multivariable model identified knowledge that pigs get infected by eating dirty feed as a significant predictor of PCC animal-level seropositivity (*p* = 0.005), OR 5.5 (95% CI 0.7–43.8) (Table 6).

**Table 5:** Risk factors associated with household-level seroprevalence of porcine cysticercosis based on univariable logistic regression with village as a random effect

Variable/category	Levels	Odds ratio (95% CI)	*p* value
District	Kamuli		
	Hoima	5 (1.3–18.8)	0.017*
Feeding pigs on yam leaves	No		
	Yes	3.4 (0.9–13.4)	0.082
Consume pork with raw vegetables	No		
	Yes	0.3 (0.1–1.1)	0.066
Knowledge that pigs get infected by eating dirty feed	No		
	Yes	6.1 (1.4–27.6)	0.018*
Infection with any GI parasite	Negative		
	Positive	0.3 (0.1–1.5)	0.139

*Significance level at *p* = 0.05.

**Table 6:** Final model of household-level risk factors for porcine cysticercosis on GLMM analysis

Variables	Category	Odds ratio (95% CI)	*p* value
Consumption of pork with raw vegetables	No		
	Yes	0.3 (0.0–2.4)	0.078
Knowledge that pigs get infected by eating dirty feed	No		
	Yes	5.5 (0.7–43.8)	0.005**

*Significance level at *p* = 0.05.

## Discussion

### Prevalence of porcine cysticercosis

Several other studies reporting prevalence and risk factors of PCC and GI parasites separately exist for different parts of Uganda. However, to the best of our knowledge, this is the first study to report the co-infection of pigs with GI parasites and PCC to guide integrated control of both parasites. The overall apparent prevalence of PCC across the two districts (Kamuli and Hoima) was found to be 4.8% (95% CI 2.7–7.1) at the individual level and 9.7% (95% CI 5.5–14.4) at the household level. The individual level prevalence is similar to that reported by Kungu et al. (2017a) for rural settings in Uganda which was 7.8% by HP10 ELISA and 3.0% by Apdia ELISA. This was below prevalences reported in other regions of Africa. In Mozambique, Pondja et al. (2010) reported 34.9%, Pouedet et al. (2002) reported 11% in Cameroon, and Shongwe et al. (2020) reported 7% in South Africa. All studies used the B158/B60 Ag ELISA. In western Kenya, Eshitera et al. (2012) reported a prevalence of 32.8% using HP10 Ag ELISA. Although all the studies used Ag ELISA, we acknowledge that different ELISA cut-off points may have been used.

Although no carcass dissection was conducted as recommended by Lightowlers et al. (2016) to rule out co-infection with *T. hydatigena* in the current study, recent studies have reported no co-infection of pigs with *T. solium* and *T. hydatigena* (Braae et al., 2015a; Kabululu et al. 2020) in similar endemic settings. This could be partly attributed to density-dependent immune-mediated interactions which have been shown to prevent co-infection of pigs with both parasites (Conlan et al. 2009). However, for monitoring *T. solium* cysticercosis control intervention outcomes, Ag ELISA should not be relied upon alone due to the false positives which may result (Kabululu et al. 2020). Additionally, the prevalence of *T. hydatigena* in dogs (the definitive host for the parasite) has not been studied in Uganda. Therefore, the possibility of cross-reactivity cannot be completely ruled out.

Hoima district had a higher prevalence of PCC at the individual level compared to Kamuli district. The apparent prevalence in Kamuli was significantly lower than that previously reported by Nsadha et al. (2014) who reported a prevalence level of 28.1% (*n* = 63) by HP10 Ag ELISA. The sensitivity of this method is 89.5% and specificity of 74% (Thomas et al. 2016; Porphyre et al. 2016). The low specificity of ELISA tests used may have resulted in high levels of false positives, and the apparent prevalence determined by HP10 ELISA seems to be often higher than those by B158/B60 Ag ELISA (now commercialized by ApDia) (Waiswa et al. 2009). This ELISA test has a sensitivity of 86.7% and specificity of 94.7% (Dorny et al. 2004) and was used in the current study.

In a study in 2005 in Kamuli and Kaliro district, Waiswa et al.,(2009) reported a B158/B60 Ag ELISA prevalence of 8.5% (95% CI 6–11) (*n* = 513 for the two districts). The drivers for the fluctuation in the prevalence rates between the 2005 study and the current study is not yet known. However, we may hypothesize this marked reduction in prevalence between the two studies could be attributed to the ongoing improvements in the pig husbandry practices, increased latrine coverage across Kamuli or adoption of MDA programs targeting neglected tropical diseases (NTDs). This finding is supported by a report of Uganda’s Ministry of Health documenting that MDA programs are annually conducted in Kamuli and Hoima districts using praziquantel to control schistosomiasis (bilharzia) (MoH 2010). Praziquantel is also effective against taeniasis in humans. This may have reduced the number of tapeworm carriers, consequently reducing PCC incidence.

In 2005, Waiswa et al. (2009) reported that free ranging management system was the most common husbandry practice in Kamuli. The shift to tethering with supplementary feeding as shown by this present study may have reduced the opportunities for pigs to have been exposed to parasite infective stages. Nsadha et al. (2010) reported rampant open defecation by farmers in the fields due to long distances to the toilets. In contrast, the current study indicates that most households (92.3%) in Kamuli district had a pit latrine, reducing the potential for transmission events. However, further studies are required to understand barriers to toilet use since the current study showed that only 42.3% of toilets had a complete wall and door, a sign of ease of use due to privacy.

Additionally, there could be differential latrine coverage across different geographical locations within the districts especially along the water bodies like Lake Albert in Hoima district and along River Nile in Kamuli district. These places are prone to flooding, and toilet construction may be challenging. Nsadha et al. (2010) noted around 25% of households lacked toilets in the large Lake Kyoga region (includes Kamuli district) and that those available were used intermittently. Seasonal confinement of pigs as observed in the current study may mean that pigs are occasionally exposed to parasite infective materials, an observation also reported by Assana et al. (2010) in Cameroon and Secka et al. (2010) in the Gambia and Senegal.

### Risk factors for porcine cysticercosis

District was a significant predictor of PCC seropositivity in the univariable analysis. The prevalence of open defecation may differ between districts, possibly because Hoima district has large areas covered with dense vegetation that may suggest ‘adequate privacy’ for open defecation. It is less so in Kamuli district. Similar observations have been made in Nigeria (Abubakar 2018) and Tanzania (Sara and Graham 2014). However, this observation needs verification by anthropological studies to explain community behaviour and its drivers on toilet use and open defecation as Thompson (2017) did.

This study identified an association between seropositivity and knowledge that pigs can get infected by eating dirty feed. In the causal diagram, we hypothesized that having knowledge regarding *T. solium* transmission would have a protective effect on seropositivity. Still, the variable appears to be a risk factor in our analysis. This relationship’s theoretical basis is unclear and may need further investigation, although we acknowledge that the association may be a statistical artefact. Other studies have found that knowledge of the transmission cycle was associated with reduced risk of the disease but not knowledge on the risk of feeding contaminated feeds (Kungu et al. 2017a). These findings may mean knowledge does not always translate to change in practice similar to observation by Sarti and Rajshekhar (2003) and Gabriël et al. (2017). Although pig farmers may know the health risk of feeding pigs dirty or contaminated feeds, resource constraints and the reliance on crop residues and swill as feed for pigs may mean infective materials are introduced to the pigs even when they are confined or tethered. Additionally, some changes in practices need to be accompanied by capital investments like construction of pig pens and toilets which may lead to the lack of change in practice even after knowledge uptake in regions with limited resources (Sarti and Rajshekhar 2003). Therefore, innovative ways of supporting change of practice may be necessary, for example, nudges (reminders), incentives and dis-incentives to reinforce good practices and deter negative practices and infrastructural investments to support change in practices.

### Prevalence and infection intensity of gastrointestinal parasites

Several studies have investigated the prevalence and intensity of pig gastrointestinal helminths in Uganda, but none has been done in Hoima district. In the current study, the overall prevalence of infection with the GI parasites was 91.8% (95% CI 88.9–94.9%) for the two districts. Kamuli district had 93.0 (95% CI 89.5–97.0) and Hoima district 90.5 (95% CI 86.1–95.0). The most prevalent infection was strongyles at 79.0% (74.3–83.6). Similar infection levels have been reported in different regions of Uganda (Nissen et al. 2011; Lagu et al. 2017;Waiswa et al. 2007).. Roesel et al. (2017) reported similar trends (61.4%; 95% CI 58.2–64.4) with strongyles being the most prevalent infection. Similar trends of parasitic infections have been reported in the neighbouring countries by Obonyo et al. (2012) and Nganga et al. (2008) in Kenya and Kabululu et al. (2015) in Tanzania. Incidentally, one pig was found to be infected with *Moniezia spp*., which is known to be a ruminant parasite but has been previously found in pigs in Peru (Gómez-Puerta et al. 2008).

The intensity of infections was also high as indicated by the high EPG counts with strongyles having the highest EPG (616.1). Nissen et al. (2011) in a study in Kabale district, Uganda, reported mean EPG for strongyles of 964 and high mean EPG for *Ascaris* spp. (4673) and *Trichuris suis* (264) than the current study. Similar intensities of parasite infections have also been reported by Lagu et al. (2017) and Waiswa et al. (2007). There was also a high OPG mean for coccidia (2042.2). Internal pig parasites are of high importance to farmers due to the resultant reduction in performance and financial losses due to their infection. Coccidiosis, particularly *Isospora suis*, cause diarrhoea and reduced growth in piglets leading to financial losses and an increase in the cost of managing the infections (Ózsvári 2018). Although deworming is widely practiced by farmers as observed in this study (97.3% in Kamuli and 98.5% in Hoima deworm pigs), the frequency may be low and the type of anthelmintic drug wrong for the existing infections leading to persistence in the infections. Forty-eight per cent of the farmers said that they dewormed the pigs after 3-month intervals, mostly using levamisole or albendazole. However, it has also been noted that farmers rely on the veterinary officer advice on what drug to administer.

### Opportunity for the integration of porcine cysticercosis and gastrointestinal nematode control

The high proportion of co-infection of pigs with GI parasites and PCC (57.4%) is of significance to report in the current study. It presents an opportunity to use integrated approaches to control both parasites. Farmers in Uganda recognize infection with worms as a major constraint to pig production (Dione et al. 2014) but not so for PCC. Farmers have also been found to extensively practice deworming of pigs to control internal parasites. We must capitalize on the ‘added value’ of control options as we investigate acceptable and sustainable interventions to control *T. solium* cysticercosis. In the current study, 95% of farmers dewormed pigs mainly using albendazole, findings similar to results by Dione et al. (2014) who reported 93% dewormed pigs mostly with ivermectin; 85% of farmers used albendazole (Kungu et al., 2017b). The anthelmintic drugs, albendazole, levamisole and ivermectin, reported to be commonly used by farmers have been found not to be effective against PCC (Mkupasi et al. 2013b, a). Promotion of regular deworming using appropriate anthelmintic and including oxfendazole as part of the deworming regime can help control GI parasite infections while at the same time controlling PCC infections. Administration of oxfendazole at 30 mg per kg effectively kills *T. solium* cysts and has been demonstrated to also control *A. suum*, *Oesophagostomum* spp*.*, *T. suis* and *Metastrongylus* spp*.* in pigs (Alvarez et al. 2013; Mkupasi et al. 2013a). However, the cost–benefit of using oxfendazole to target GI worms while controlling PCC needs to be investigated.

### Limitation of the study

The current study was embedded within the Uganda Pig Genetics project that was designed as a longitudinal study. The implementation of a cross-sectional study using the same study subjects may have biased the risk factor analysis since they had been in touch with research teams during data collection and may have improved their knowledge, perception and even practices on pig husbandry. We used ApDia ELISA kits to detect *Taenia solium* circulating antigens in pig serum, but there is known cross-reactivity with *Taenia hydatigena* antigens that may raise false positives. We had planned to carry out carcass dissection which is the ‘gold standard’ technique on all the seropositive pigs, but this was not possible due to the COVID-19 pandemic which prevented the team from returning for farm visits to procure the positive pigs before they were sold for slaughter. For estimation of GI parasite infections, we did not reach the estimated sample size since some of the targeted farmers had ineligible or had sold their pigs. However, the results are robust since the prevalence of the GI parasites was high.

## Conclusion

This study provides data on the current epidemiological status of PCC and pig GI parasites in Kamuli and Hoima districts, Uganda. Our findings demonstrate that *T. solium* cysticercosis in pigs is more prevalent in Hoima district than in Kamuli district which was lower than previously reported. Knowledge that pigs can get infected by eating dirt feeds a significant predictor for *T. solium cysticercosis* seropositivity at the household level. The prevalence of infection with gastrointestinal parasites was high and similar across the two districts. There is also a high likelihood of pigs being infected with both PCC and GI parasites. Since deworming is practiced by many farmers in the study districts, the high rate of co-infection presents an opportunity for integrated control using an anthelmintic capable of eliminating both *T. solium cysts* and other pig worms. Further studies are required to identify and test the feasibility, cost–benefit analysis and acceptability of using such anthelmintics (including oxfendazole).

## Supplementary Information

Below is the link to the electronic supplementary material.

Supplementary file1 (DOCX 16 KB)

## Data Availability

All relevant data are within the paper and its supporting information files.

## References

[CR1] Abubakar IR (2018). Exploring the determinants of open defecation in Nigeria using demographic and health survey data. Sci Total Environ.

[CR2] Alvarez L, Saumell C, Fusé L (2013). Efficacy of a single high oxfendazole dose against gastrointestinal nematodes in naturally infected pigs. Vet Parasitol.

[CR3] ApDia (2019) Cysticercosis. http://www.apdiagroup.com/ct-menu-item-11/elisa/2014-04-15-19-55-49/cysticercosis-ag-elisa. Accessed 23 May 2019

[CR4] Assana E, Amadou F, Thys E (2010). Pig-farming systems and porcine cysticercosis in the north of Cameroon. J Helminthol.

[CR6] Babigumira BM, Sölkner J, Mészáros G (2021). A mix of old British and modern European breeds: genomic prediction of breed composition of smallholder pigs in Uganda. Front Genet.

[CR7] Barto K (2020) Multi-model inference

[CR8] Bates DM (2010). lme4: mixed-effects modeling with R.

[CR9] Braae UC, Kabululu M, Nørmark ME (2015). Taenia hydatigena cysticercosis in slaughtered pigs, goats, and sheep in Tanzania. Trop Anim Health Prod.

[CR10] Braae UC, Saarnak CFL, Mukaratirwa S (2015). Taenia solium taeniosis/cysticercosis and the co-distribution with schistosomiasis in Africa. Parasit Vectors.

[CR11] Conlan JV, Vongxay K, Fenwick S (2009). Does interspecific competition have a moderating effect on Taenia solium transmission dynamics in Southeast Asia?. Trends Parasitol.

[CR12] Dione MM, Ouma EA, Roesel K (2014). Participatory assessment of animal health and husbandry practices in smallholder pig production systems in three high poverty districts in Uganda. Prev Vet Med.

[CR13] Dorny P, Phiri IK, Vercruysse J (2004). A Bayesian approach for estimating values for prevalence and diagnostic test characteristics of porcine cysticercosis. Int J Parasitol.

[CR14] Eshitera EE, Githigia SM, Kitala P (2012). Prevalence of porcine cysticercosis and associated risk factors in Homa Bay District. Kenya BMC Vet Res.

[CR15] FAOSTAT (2021) Pig production: livestock primary data, Uganda. http://www.fao.org/faostat/en/#data/QL/visualize. Accessed 30 Apr 2021

[CR16] Gabriël S, Dorny P, Mwape KEE (2017). Control of Taenia solium taeniasis/cysticercosis: the best way forward for sub-Saharan Africa?. Acta Trop.

[CR17] García HH, Gonzalez AE, Evans CAWW (2003). Taenia solium cysticercosis. Lancet (London, England).

[CR18] Gómez-Puerta LA, Lopez-Urbina MT, González AE (2008). Occurrence of Moniezia expansa (Rud, 1810) Blanchard, 1891 (Cestoda: Anoplocephalidae) in domestic pig (Sus scrofa domestica Linnaeus, 1758) in Perú. Vet Parasitol.

[CR19] Havelaar AH, Kirk MD, Torgerson PR (2015). World Health Organization global estimates and regional comparisons of the burden of foodborne disease in 2010. PLOS Med.

[CR20] Kabululu ML, Johansen MV, Mlangwa JED (2020). Performance of Ag-ELISA in the diagnosis of Taenia solium cysticercosis in naturally infected pigs in Tanzania. Parasit Vectors.

[CR21] Kabululu ML, Ngowi HA, Kimera SI (2015). Risk factors for prevalence of pig parasitoses in Mbeya Region, Tanzania. Vet Parasitol.

[CR22] Kipper M, Andretta I, Monteiro SG (2011). Meta-analysis of the effects of endoparasites on pig performance. Vet Parasitol.

[CR23] Knecht D, Popiołek M, Zaleśny G (2011). Does meatiness of pigs depend on the level of gastro-intestinal parasites infection?. Prev Vet Med.

[CR24] Kungu JM, Dione M, Ejobi F (2017). Risk factors, perceptions and practices associated with Taenia solium cysticercosis and its control in the smallholder pig production systems in Uganda: a cross-sectional survey. BMC Infect Dis.

[CR25] Kungu JM, Dione MM, Ejobi F (2017). Sero-prevalence of Taenia spp. cysticercosis in rural and urban smallholder pig production settings in Uganda. Acta Trop.

[CR26] Kungu JM, Dione MM, Ocaido M, Ejobi F (2015) Status of Taenia solium cysticercosis and predisposing factors in developing countries involved in pig farming. Int J One Heal Available www.onehealthjournal.org Int J One Heal 1:. 10.14202/IJOH.2015.6-13

[CR27] Lagu C, Andama M, Lee S, et al (2017) Prevalence and intensity of internal parasites in pigs under indigenous micro-organism (IMO) and conventional piggery farms, greater Mbarara, Uganda. Livest Res Rural Dev 29:

[CR28] Lightowlers MW, Garcia HH, Gauci CG (2016). Monitoring the outcomes of interventions against Taenia solium: options and suggestions. Parasite Immunol.

[CR29] MAAIF and UBOS (2008) The national livestock census a summary report of the national livestock census. Uganda Bur Stat May:1–34

[CR30] Mkupasi EM, Ngowi HA, Sikasunge CS (2013). Efficacy of ivermectin and oxfendazole against Taenia solium cysticercosis and other parasitoses in naturally infected pigs. Acta Trop.

[CR31] Mkupasi EM, Sikasunge CS, Ngowi HA, Johansen MV (2013). Efficacy and safety of anthelmintics tested against Taenia solium cysticercosis in pigs. PLoS Negl Trop Dis.

[CR32] MoH (2010) Uganda health sector strategic and investment plan III (HSSIP).

[CR33] Nejsum P, Betson M, Bendall RP, et al (2012) Assessing the zoonotic potential of Ascaris suum and Trichuris suis: looking to the future from an analysis of the past. In: Journal of Helminthology. J Helminthol, pp 148–155 10.1017/S0022149X12000193 22423595

[CR34] Nganga CJ, Karanja DN, Mutune MN (2008). The prevalence of gastrointestinal helminth infections in pigs in Kenya. Trop Anim Health Prod.

[CR35] Nissen S, Poulsen IH, Nejsum P, et al (2011) Prevalence of gastrointestinal nematodes in growing pigs in Kabale District in Uganda. 567–572. 10.1007/s11250-010-9732-x 10.1007/s11250-010-9732-x 21088893

[CR36] Nsadha Z, Saimo M, Waiswa C (2010). Risk factors and lingual prevalence of porcine cysticercosis in the Lake Kyoga Basin in Uganda. Africa J Anim Biomed Sci.

[CR37] Nsadha Z, Thomas LF, Fèvre EM (2014). Prevalence of porcine cysticercosis in the Lake Kyoga Basin. Uganda BMC Vet Res.

[CR38] Obonyo FO, Maingi N, Githigia SM, Ng’ang’a CJ (2012) Prevalence, intensity and spectrum of helminths of free range pigs in Homabay district, Kenya. Livest Res Rural Dev 24:

[CR39] Ouma E, Ochieng J, Dione M, Pezo D (2017) Governance structures in smallholder pig value chains in Uganda: constraints and opportunities for upgrading. Int Food Agribus Manag Rev 20:307–319. 10.22434/IFAMR2014.0176

[CR40] Ouma EA, Dione MM, Lule P, et al (2015) Smallholder pig value chain assessment in Uganda: results from producer focus group discussions and key informant interviews. 1–141

[CR41] Ouma EA, Dione MM, Lule PM, et al (2014) Characterization of smallholder pig production systems in Uganda: constraints and opportunities for engaging with market systems. Livest Res Rural Dev

[CR42] Ózsvári L (2018) Production impact of parasitisms and coccidiosis in swine. J Dairy, Vet Anim Res 7:. 10.15406/jdvar.2018.07.00214

[CR43] Phiri IK, Ngowi H, Afonso S (2003). The emergence of Taenia solium cysticercosis in Eastern and Southern Africa as a serious agricultural problem and public health risk. Acta Trop.

[CR44] Pondja A, Neves L, Mlangwa J (2010). Prevalence and risk factors of porcine cysticercosis in Angónia District. Mozambique PLoS Negl Trop Dis.

[CR45] Porphyre V, Betson M, Rabezanahary H (2016). Taenia solium porcine cysticercosis in Madagascar: comparison of immuno-diagnostic techniques and estimation of the prevalence in pork carcasses traded in Antananarivo city. Vet Parasitol.

[CR46] Pouedet MSR, Zoli AP, Nguekam,  (2002). Epidemiological survey of swine cysticercosis in two rural communities of West-Cameroon. Vet Parasitol.

[CR47] Roesel K, Dohoo I, Baumann M (2017). Prevalence and risk factors for gastrointestinal parasites in small-scale pig enterprises in Central and Eastern Uganda. Parasitol Res.

[CR48] Sara S, Graham J (2014). Ending open defecation in rural Tanzania: which factors facilitate latrine adoption?. Int J Environ Res Public Health.

[CR49] Sarti E, Rajshekhar V (2003). Measures for the prevention and control of Taenia solium taeniosis and cysticercosis. Acta Trop.

[CR50] Scare JA, Slusarewicz P, Noel ML (2017). Evaluation of accuracy and precision of a smartphone based automated parasite egg counting system in comparison to the McMaster and mini-FLOTAC methods. Vet Parasitol.

[CR51] Secka A, Marcotty T, De Deken R (2010). Porcine cysticercosis and risk factors in The Gambia and Senegal. J Parasitol Res.

[CR52] Shongwe NA, Byaruhanga C, Dorny P (2020). Knowledge, practices and seroprevalence of Taenia species in smallholder farms in Gauteng. South Africa PLoS One.

[CR53] Signorell A (2020) Tools for descriptive statistics [R package DescTools version 0.99.39]

[CR54] Textor J, Hardt J, Knüppel S (2011). DAGitty Epidemiology.

[CR55] Thomas LF, Harrison LJS, Toye P (2016). Prevalence of Taenia solium cysticercosis in pigs entering the food chain in western Kenya. Trop Anim Health Prod.

[CR56] Thompson R (2017) Pigs, people, pathogens: a qualitative analysis of the pig value chain in the central region of Uganda. International Livestock Research Institute

[CR57] Twine R, Njehu A (2020) Uganda smallholder pig value chain development : situation analysis and trends. Nairobi, Kenya

[CR58] Uganda Bureau of Statistics (2016) National population and housing census 2014. Kampala

[CR59] Uganda Bureau of Statistics (2018) Uganda Bureau of Statistics: statistical abstract

[CR60] Waiswa C, Fèvre EM, Nsadha Z (2009). Porcine cysticercosis in Southeast Uganda: seroprevalence in Kamuli and Kaliro Districts. J Parasitol Res.

[CR61] Waiswa C, Mubwoli J, Wampande E, Oweikanga JK (2007). Prevalence of endoparasitic infections in pigs of South Eastern Uganda. Africa J Anim Biomed Sci.

[CR62] Warnes AGR, Bolker B, Lumley T, Randall C (2018) Package ‘ gmodels ’

[CR63] Zajac A, Conboy G (2007) Veterinary clinical parasitology, 8th Editio. Wiley-Blackwell, Iowa

[CR64] Zimmerman JJ, Karriker LA, Ramirez A, et al (2012) Diseases of swine, 10th edn. Wiley-Blackwell, Chichester, West Sussex, PO19 8SQ, UK

[CR65] Zoli A, Shey-Njila O, Assana E (2003). Regional status, epidemiology and impact of Taenia solium cysticercosis in Western and Central Africa. Acta Trop.

