# Supplementary material for: Co-infection of pigs with Taenia solium cysticercosis and gastrointestinal parasites in Eastern and Western Uganda
Source: Parasitol Res. 2021 Nov 24;121(1):177–89. doi: 10.1007/s00436-021-07380-9 (PMC8610610; doi:10.1007/s00436-021-07380-9)
Supplement: Supplementary file 1 — Supplementary file1 (DOCX 16 KB) [file 436_2021_7380_MOESM1_ESM.docx]

**Supplementary material:** **univariate regression table**

**Table 7: Variables tested for association with PCC seropositivity based on univariable logistic regression with village as a random effect**

| **Variable/category** | **Levels** | **Odds ratio (95% CI)** | **P value** |
| --- | --- | --- | --- |
| District | Kamuli | 1 (ref) |  |
|  | Hoima | 5 (1.3 – 18.8) | 0.017* |
| Sex of the respondent | Male | 1(ref) |  |
|  | Female | 1.7 (0.5 – 6.3) | 0.433 |
| Feeding pigs on yam leaves | No | 1 (ref) |  |
|  | Yes | 3.4 (0.9 – 13.4) | 0.082 |
| Feeding pigs on unboiled swill | No | 1 (ref) |  |
|  | Yes | 0.6 (0.2 – 1.9) | 0.364 |
| Deworming pigs | No | 1 (ref) |  |
|  | Yes | 0.2 (0.3 – 3.3) | 0.262 |
| Consumption of pork | No | 1 (ref) |  |
|  | Yes | 0.6 (0.1 – 3.8) | 0.594 |
| Consume pork with raw vegetables | No | 1 (ref) |  |
|  | Yes | 0.3 (0.1 – 1.1) | 0.066 |
| Knowledge that pigs get infected by eating dirt feed | No | 1 (ref) |  |
|  | Yes | 6.1 (1.4– 27.6) | 0.018* |
| Water for pigs from shallow well | No | 1 (ref) |  |
|  | Yes | 2.7 (0.4 – 18.1) | 0.309 |
| Infection with any GI parasite | Negative | 1(ref) |  |
|  | Positive | 0.3 (0.1 – 1.5) | 0.139 |
| Polyparasitism | Negative | 1 (ref) |  |
|  | Positive | 0.6 (0.2 – 2.0) | 0.363 |

*Significance level at p=0.05
